# How Zebrafish Can Drive the Future of Genetic-based Hearing and Balance Research

**DOI:** 10.1007/s10162-021-00798-z

**Published:** 2021-04-28

**Authors:** Lavinia Sheets, Melanie Holmgren, Katie S Kindt

**Affiliations:** 1 Department of Otolaryngology–Head & Neck Surgery, Washington University School of Medicine, St. Louis, MO, USA; 2 Section On Sensory Cell Development and Function, National Institutes On Deafness and Other Communication Disorders, National Institutes of Health, Bethesda, USA

**Keywords:** zebrafish, hearing and balance, genetics, genetic screening

## Abstract

Over the last several decades, studies in humans and animal models have successfully identified numerous molecules required for hearing and balance. Many of these studies relied on unbiased forward genetic screens based on behavior or morphology to identify these molecules. Alongside forward genetic screens, reverse genetics has further driven the exploration of candidate molecules. This review provides an overview of the genetic studies that have established zebrafish as a genetic model for hearing and balance research. Further, we discuss how the unique advantages of zebrafish can be leveraged in future genetic studies. We explore strategies to design novel forward genetic screens based on morphological alterations using transgenic lines or behavioral changes following mechanical or acoustic damage. We also outline how recent advances in CRISPR-Cas9 can be applied to perform reverse genetic screens to validate large sequencing datasets. Overall, this review describes how future genetic studies in zebrafish can continue to advance our understanding of inherited and acquired hearing and balance disorders*.*

## INTRODUCTION

Zebrafish were established as a model organism to study vertebrate development and gene function in the 1970s by George Streisinger and colleagues (Kimmel 1989; Streisinger et al. 1981). Subsequent forward genetic screens put zebrafish on the map and established it as a valuable vertebrate genetic model system (Driever et al. 1996; Haffter et al. 1996; Nüsslein-Volhard 2012). Today, large-scale mutagenic screens in zebrafish remain advantageous because they can be high-throughput and cost-effective relative to rodent models. For example, a large number of adult zebrafish can be housed together inexpensively compared to rodents. Furthermore, from a single spawning, an adult pair can produce hundreds of embryos to screen (Dahm and Geisler 2006; Lawrence 2007). There are a variety of additional reasons zebrafish are a popular model organism for research. With regard to hearing and balance, zebrafish are advantageous because they develop externally and are transparent through embryonic and larval stages. This enables in vivo access for experimentation and observation (Whitfield et al. 2002). This accessibility is an extremely important advantage in the context of the auditory and vestibular system, as hair cell epithelia in mammals are encased in the temporal bone of the skull, making them challenging to access in vivo.

This in vivo access has been especially beneficial for developmental studies because zebrafish embryos develop extremely fast. The hair cell epithelia of zebrafish begin to form within 24 h after fertilization (McGraw et al. 2017; Schneider-Maunoury and Pujades 2007). This rapid developmental trajectory leads to the formation of hair cell sensory systems that are functional just 5 days post fertilization (dpf) (Kimmel et al. 1974; Oteiza et al. 2017; Suli et al. 2012). Research in zebrafish has been augmented by leveraging its genetic tractability to create transgenic lines expressing fluorescent indicators and by developing fluorescent dye labeling approaches (Kwan et al. 2007). Together these tools have strengthened researchers ability to visualize and study hair cell sensory systems in zebrafish (Behra et al. 2012; Fetcho and O’Malley 1995; Harris et al. 2003; Lacoste et al. 2015; Obholzer et al. 2008; Seiler and Nicolson 1999; Trapani et al. 2009). These types of analyses have been useful not only for developmental studies but also for studies of cellular function and mechanisms of disease. Moreover, the ability to access structures in toto has been beneficial for electrophysiological and imaging-based activity measurements (Migault et al. 2018; Nicolson et al. 1998; Tabor et al. 2019; Vanwalleghem et al. 2020; Zhang et al. 2018).

**Fig. 1:**
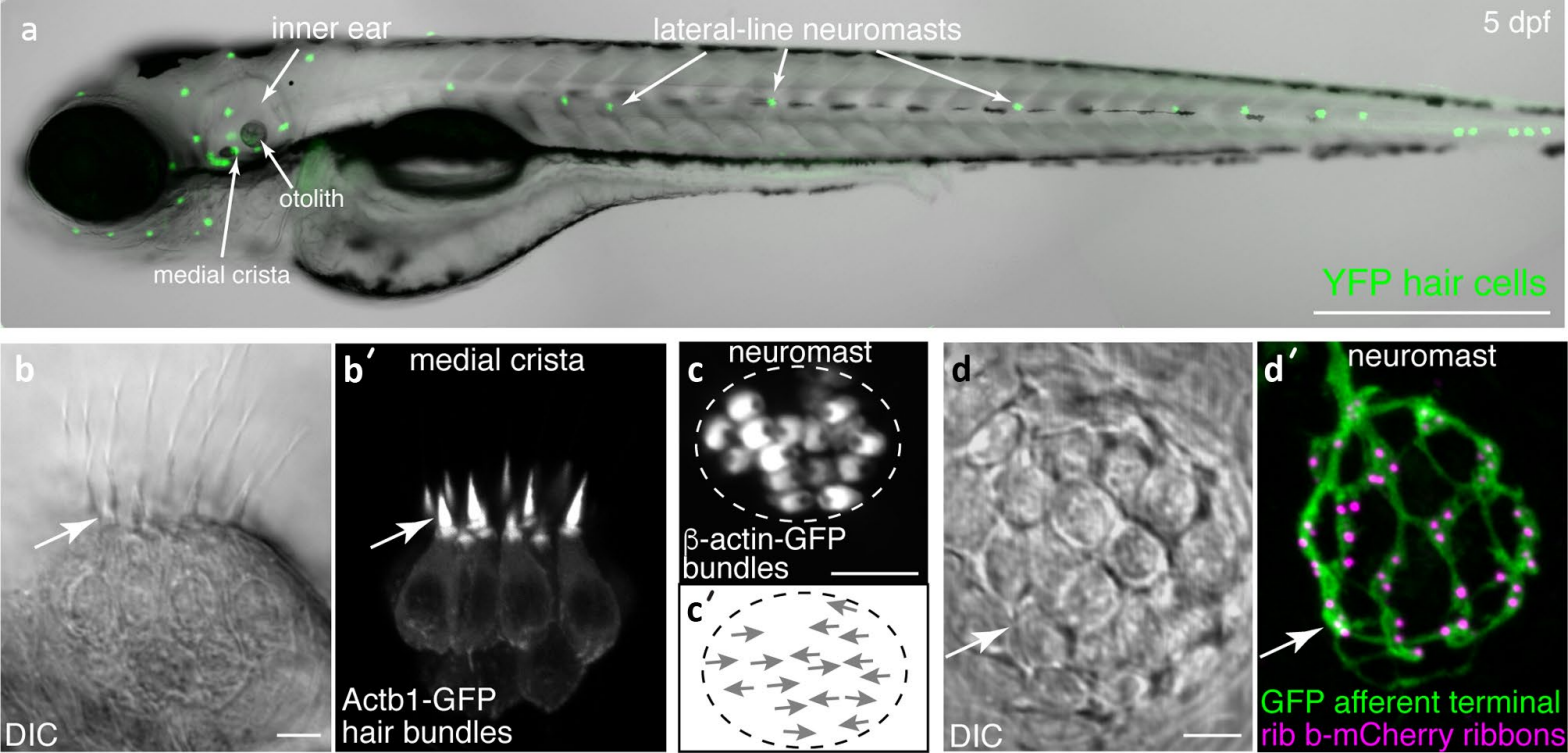
Visualization of structures in hair cell systems of larval zebrafish. **(a)** A zebrafish larva at 5 days post fertilization (dpf) is shown. At this stage, both the inner ear and lateral line hair cell systems are functional. In this transgenic larvae, all hair cells are visualized via YFP fluorescence (*Tg[myo6b:D3cpv]*^*vo9*^ (Kindt et al. 2012)). (**b**–**b′**) High magnification, side-view of hair cells in the medial crista (inner ear). A DIC image **(b)** and corresponding fluorescence image shows hair cells expressing β-actin-GFP to visualize hair bundles (**b′**, *Tg(myo6b:actb1-EGFP)*^*vo8*^ (Kindt et al. 2012)). (**c**–**c′**) High magnification, top-down image of a neuromast hair cells expressing β-actin-GFP (**c′**, *Tg(myo6b:actb1-EGFP)*^*vo8*^) can also reveal hair bundle orientations (**c′**, arrows indicate orientations). (**d**–**d′**) DIC image **(d)** and corresponding fluorescence image shows hair cells expressing Rib b-mCherry to label hair cell ribbons and afferent neurons expressing GFP to label the innervating fiber (**d′**, *Tg(myo6b:ctbp2l-mCherry)*^*idc3*^; *Tg(neurod1:EGFP)*^*nl1*^ (Sheets et al. 2017; Trapani et al. 2009). The image in a was taken at × 10, while all other images were taken at × 63 magnification. All images were taken at 5 dpf. Scale bar = 500 µm in **a** and 5 µm in **b**, **c**, and **d**

All these valuable approaches have been applied to study the two hair cell sensory systems in zebrafish, the inner ear and the lateral line. The zebrafish inner ear (Fig. 1a) is required for proper hearing and balance. The lateral line system is made up of superficial clusters of hair cells called neuromasts that are distributed along the surface of the fish (Fig. 1a). The lateral line system is specialized for detecting fluid flow, and it is important for many behaviors including shoaling, feeding, and evading predators (Faucher et al. 2010; McHenry et al. 2009; Mekdara et al. 2018; Olszewski et al. 2012; Suli et al. 2012). Similar to mammals, in zebrafish larvae, the inner ear contains 3 semicircular canals with associated cristae (e.g.: Fig. 1b), and two maculae homologous to the mammalian utricle and saccule. In zebrafish, maculae are associated with otoliths (Fig. 1a), calcium carbonate stones that are essential to effectively transduce sensory stimuli (Inoue et al. 2013; Yao et al. 2016). In adult and larval zebrafish, the utricular macula is essential for balance and detecting gravity. When the utricle is disrupted, zebrafish fail to sense gravity and lack vestibulo-oculomotor behaviors (Kwak et al. 2006; Mo et al. 2010; Riley and Moorman 2000). In larval zebrafish, the saccular macula detects sound (Yao et al. 2016), while in adult zebrafish, sound is detected via the saccule and another macula, the lagena (Fay and Popper 2000; Ladich and Schulz-Mirbach 2016). The use of the saccule (and lagena in adults) to detect sound is a major difference between zebrafish and mammals, where a specialized auditory organ, the cochlea, is used to detect sound (Manley 2012). Zebrafish inner ear hair cells send signals directly to afferent neurons of the VIII nerve or statoacoustic ganglion (Andermann et al. 2002; Whitfield et al. 2002), whereas lateral line hair cells send signals directly to afferent neurons in the anterior or posterior lateral-line ganglia (aLLg and pLLg) (Alexandre and Ghysen 1999).

Early forward genetic screens revealed that zebrafish could be used to identify genes essential for hearing and balance. These screens were performed in larvae and identified mutants based on morphological or behavioral defects (Granato et al. 1996; Malicki et al. 1996; Nicolson et al. 1998; Whitfield et al. 1996). Morphologically, mutants were assessed visually in vivo—identifying mutants with alterations to the otic vesicle, semicircular canals, or hair cell epithelia. Behaviorally, mutants with impaired hair cell system function were assessed based on acoustic startle responses and motility. Mutants with defects in hearing and balance fail to startle in response to acoustic stimuli and swim in circles. Identification of the lesions underlying these morphological and behavioral defects in these mutants revealed a striking conservation of genes. The same genes, when mutated, resulted in hearing and balance defects in zebrafish, mice, and humans (reviewed in Nicolson 2005). This is consistent with conservation at the genome level, as approximately 70 % of human genes have at least one clear zebrafish ortholog (Howe et al. 2013).

Collectively, forward genetic screens in zebrafish expanded our knowledge of the molecular basis of hearing and balance. Despite the value of these screens, they come with limitations. For example, not all regions in the genome are lesioned equally by the mutagens used in these screens, such as genes with a relatively small genomic footprint. Additionally, some molecules required for hearing and balance may not have been identified due to severe morphological defects or lethality associated with the gene lesion. Another limitation of forward genetic screens in zebrafish is the result of a whole genome duplication event in the teleost lineage after it diverged from tetrapods (Postlethwait et al. 2000). While some gene duplicates were lost over time, it is estimated that 20 % of duplicated gene pairs were retained (Postlethwait et al. 2000). When duplicates are retained, there can be two paralogs for a single mammalian gene and lesioning just one paralog may not be sufficient to eliminate gene function. Information regarding zebrafish genes and gene duplicates can be found on the Zebrafish Information Network (ZFIN): https://zfin.org/.

Regardless of these challenges, forward genetic screens in zebrafish have paved a powerful path for gene discovery. This path has been complemented by reverse genetic approaches which have allowed for more targeted ways to lesion genes, or gene duplicates. To date, reverse genetic approaches have produced a large collection of zebrafish mutants with unexplored function that could be important for hearing and balance (Amsterdam et al. 2011; Moens et al. 2008; Sood et al. 2006). Wildtype zebrafish and mutant zebrafish lines are available from the Zebrafish International Resource Center (ZIRC), at the University of Oregon (http://zebrafish.org/zirc/home/guide.php), and the European Zebrafish Resource Center (EZRC), at the Karlsruhe Institute of Technology (https://www.ezrc.kit.edu/).

This review discusses why zebrafish are a powerful genetic model for studying auditory and vestibular systems, with an emphasis on peripheral hair cells and sensory afferents. It provides a historic overview of genetic studies that have used zebrafish to identify and characterize genes important for hearing and balance. Later, the review expands to survey how both traditional and modern genetic methods can be used to generate new zebrafish mutants and develop new screening strategies to advance hearing and balance research. Finally, this review examines how the zebrafish model can be used to screen large genomic and transcriptomic datasets to characterize candidate human deafness genes.

## MAIN BODY

### Forward Genetic Screens to Study Hearing and Balance in Zebrafish

**Fig. 2:**
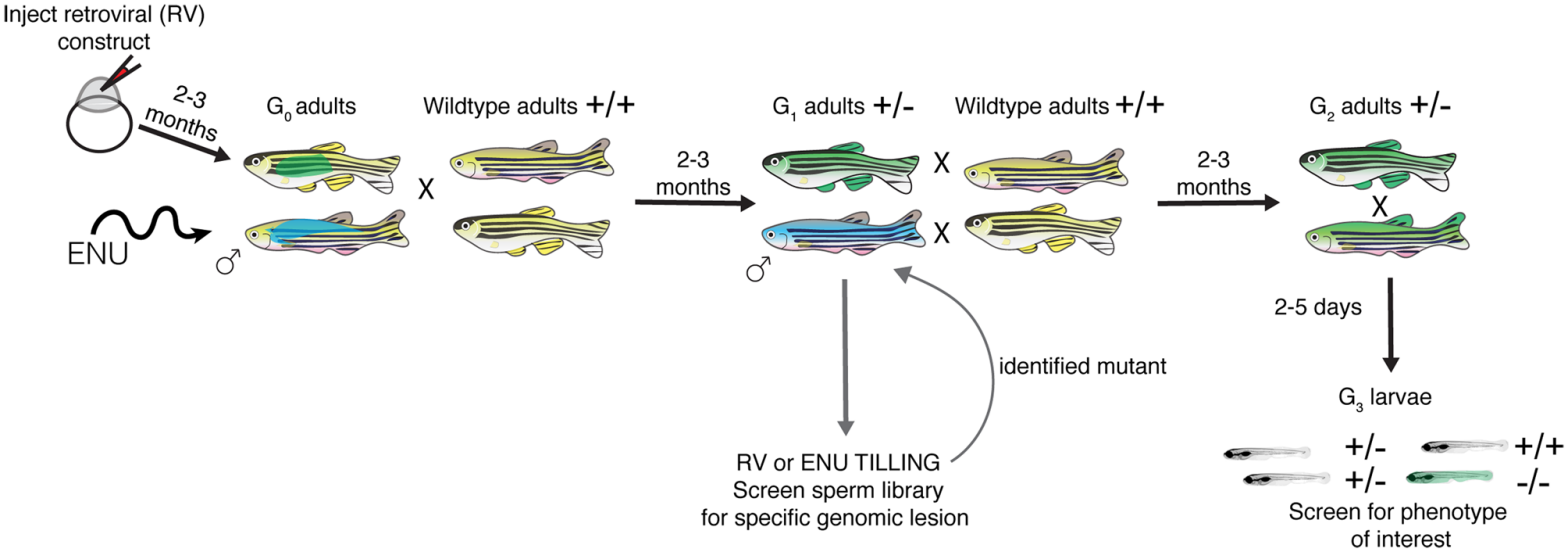
Outline of mutagenesis in zebrafish used for TILLING and forward genetic screening. Both retroviruses and the mutagen ENU can be used to create germline mutations in zebrafish. For retroviral-based mutagenesis, a DNA construct containing the retrovirus is injected into newly fertilized zebrafish embryos. These injected embryos are grown to adulthood (2–3 months), resulting in G_0_ adults that are mosaic for germline mutations. G_0_ adults are then outcrossed to wildtype adults to generate G_1_ adults that are heterozygous for different genetic lesions. In chemical mutagenesis, adult males are treated with a chemical mutagen such as ENU. These mutagenized males are crossed to wildtype adult females to generate G1 adults that are heterozygous for different genetic lesions. For a forward genetic screen (ENU or retroviral-based mutagens), G_1_ adults are crossed to wildtype animals, providing a pool of G_2_ adult carriers. G_2_ adults are incrossed, and G_3_ larvae are screened for a phenotype of interest. When mutagens are used to screen for a specific genetic lesion (in the case of TILLING), G_1_ adults or sperm stored in a library from G_1_ males are screened for mutations. After identifying a specific mutation, the identified G_1_ adult is crossed to wildtype to generate G_2_ adults harboring that mutation. Two G_2_ adults with the lesion of interest are then incrossed and screened for phenotypes. Blue and green represent distinct genetic lesions in mutagenized fish

Studies on hereditary deafness in humans, in inbred mouse populations, and forward genetic screens in mice and zebrafish have identified numerous fundamental molecules that are required for the development and function of auditory and vestibular systems across vertebrate species (Ingham et al. 2019; Nicolson et al. 1998; Schwander et al. 2007; Whitfield et al. 1996). These unbiased screens are invaluable because they begin with a screenable phenotype and then identify discrete mutations within genes that are linked to the phenotype (Fig. 2). Furthermore, identification of multiple mutant alleles can reveal specific protein motifs or residues within proteins that are essential for function.

In zebrafish, forward genetic screens are based on a three generation scheme (Fig. 2) (Patton and Zon 2001). Traditionally, forward genetic screens use the chemical *N*-ethyl-*N*-nitrosourea (ENU) to mutagenize the testes of adult male zebrafish. In the first generation, G_0_, mutagenized males are crossed to wildtype females and G_1_ larvae are grown to adulthood. Once adults, G_1_ fish are in-crossed and G_2_ progeny are grown to adulthood. The progeny of G_2_ adults (G_3_) are screened for phenotypes associated with homozygous recessive mutations at embryonic or larval stages. Later, the gene disruption associated with the phenotype is identified through genetic mapping. Other mutagens such as retroviral (RV) constructs have also been used in zebrafish. RV-based mutagenesis utilizes the random insertion of viral sequences into coding regions of genes to disrupt the open reading frame. RV constructs are injected into newly fertilized embryos; the G_0_ embryos are then grown to adulthood. G_0_ adults are crossed to wildtype animals and follow a similar three generation screening strategy (Fig. 2). A benefit to RV-based mutagenesis is a more straightforward way of mapping genetic lesions compared to chemical mutagens (Amsterdam et al. 2011). Both chemical and RV-based screens have been used to identify zebrafish mutants with behavioral (hearing and balance) and morphological (the inner ear and lateral line) defects (Gleason et al. 2009; Granato et al. 1996; Nicolson et al. 1998; Nissen et al. 2003; Pei et al. 2018; Whitfield et al. 1996). Overall, it was these forward genetic screens that established zebrafish as a viable, genetically tractable model to study hearing and balance.

#### Morphology-Based Forward Genetic Screens to Study Hair Cell Systems in Zebrafish

Due to the optical clarity of larval zebrafish and their rapid development, forward genetic screens have been widely used to identify mutants with distinct morphological phenotypes. With regard to hair cell systems, numerous mutant phenotypes have been identified, such as defects in inner ear size, otoliths (stones required for macular function), semi-circular canals, hair cell patches, and lateral line formation (Geng et al. 2013; Malicki et al. 1996; Whitfield et al. 1996). Overall, forward genetic screens in zebrafish focused on inner-ear development have been invaluable. In vivo screening during inner ear development is not possible in mammals because the mammalian inner ear is located deep in the skull and encased in bone. To date, morphology-based screens (reviewed in Whitfield 2002, and Whitfield et al. 1996) have identified many conserved genes required for inner ear formation, including molecules required for otolith development and tethering (e.g., *otogelin* and $\alpha$*-tectorin* (Stooke-Vaughan et al. 2015)) and endolymphatic fluid regulation (e.g., *slc12a2* (Abbas and Whitfield 2009)). All three of these genes play important roles in the mammalian inner ear and are associated with human hearing loss (DFNB84 (*otogelin*); DFNA8, DFNA12, and DFNB21 ($\alpha$*-tectorin*); DFNA78 (*slc12a2)*) (Mustapha et al. 1999; Mutai et al. 2020; Verhoeven et al. 1998; Yariz et al. 2012).

In addition to identifying genes required for inner-ear development, morphology-based forward genetic screens have been used in zebrafish to identify gene-environment interactions that regulate hair cell death (Hailey et al. 2012; Owens et al. 2008; Stawicki et al. 2014). Understanding how hair cells are damaged by environmental insults is clinically important as the majority of non-inherited, environmental hearing loss results from damage to or loss of hair cells (World Health Organization 2018). Several forward genetic screens have been performed to identify genes that modulate susceptibility to hair cell death following exposure to ototoxic drugs. Ototoxicity screens have largely centered on lateral line hair cells in larval zebrafish. The lateral line hair cells are advantageous for these screens because they are directly exposed to the aqueous environment, and for screening, ototoxic drugs can simply be added the media. Ototoxicity screens were designed by assaying hair cell death using vital dyes or transgenic lines that express GFP in hair cells (Hailey et al. 2012; Owens et al. 2008; Stawicki et al. 2014). Using this approach, three genes have been identified that modulate the susceptibility of hair cells to aminoglycoside-induced ototoxicity–*slc4a1b* and *gcm2*, two genes important for pH regulation (Hailey et al. 2012; Stawicki et al. 2014), and *cc2d2a*, a ciliary transition zone gene that may play a role in vestibular aminoglycoside ototoxicity (reviewed in Stawicki et al. 2015).

Extensive work has also examined the genetic underpinnings of hair cell regeneration in zebrafish. Early work has shown that in many fish species, including zebrafish, hair cells readily regenerate after damage (reviewed in Lush and Piotrowski 2014; Monroe et al. 2015). Therefore, an intriguing question in the field has been why this regenerative capability has been lost in mammals. Knowledge generated from studying hair cell regeneration in zebrafish can be used to develop regenerative therapies to restore hearing loss in humans. Consequently, forward genetic screens similar to those used to identify molecules influencing hair cell death and protection have been performed to identify molecules mediating hair cell regeneration (Behra et al. 2009). Yet to date, in unbiased forward genetic screens in larval zebrafish only one molecule, Phoenix, has been identified that impacts hair cell regeneration. *Phoenix* is a novel gene expressed in supporting cells of lateral line neuromasts and is required for hair cell regeneration in zebrafish (Behra et al. 2009). The function of Phoenix remains to be explored, and the gene has no known homolog in mammals. Currently, our understanding of the pathways underlying damage and subsequent regeneration in hair cell organs in zebrafish versus mammals is incomplete. This fundamental knowledge is required to unlock the mystery of how and why zebrafish hair cells regenerate and mammalian hair cells do not. It is possible that future genetic studies in zebrafish and other species with regenerative capabilities, such as avian or amphibian models, will help uncover these differences.

#### Transgenic Zebrafish in Morphology-Based Forward Genetic Screens to Study Hair Cell Systems

The ability to quickly and efficiently create transgenic lines in zebrafish (Kawakami 2007; Kwan et al. 2007) has facilitated more elaborate morphology-based forward genetic screens. Currently, there are a variety of transgenic zebrafish lines that fluorescently label organs, cells and subcellular structures within the zebrafish auditory, vestibular and lateral line systems. These lines can be used to perform unbiased screens to identify more subtle aspects of the auditory and vestibular system.

**Fig. 3:**
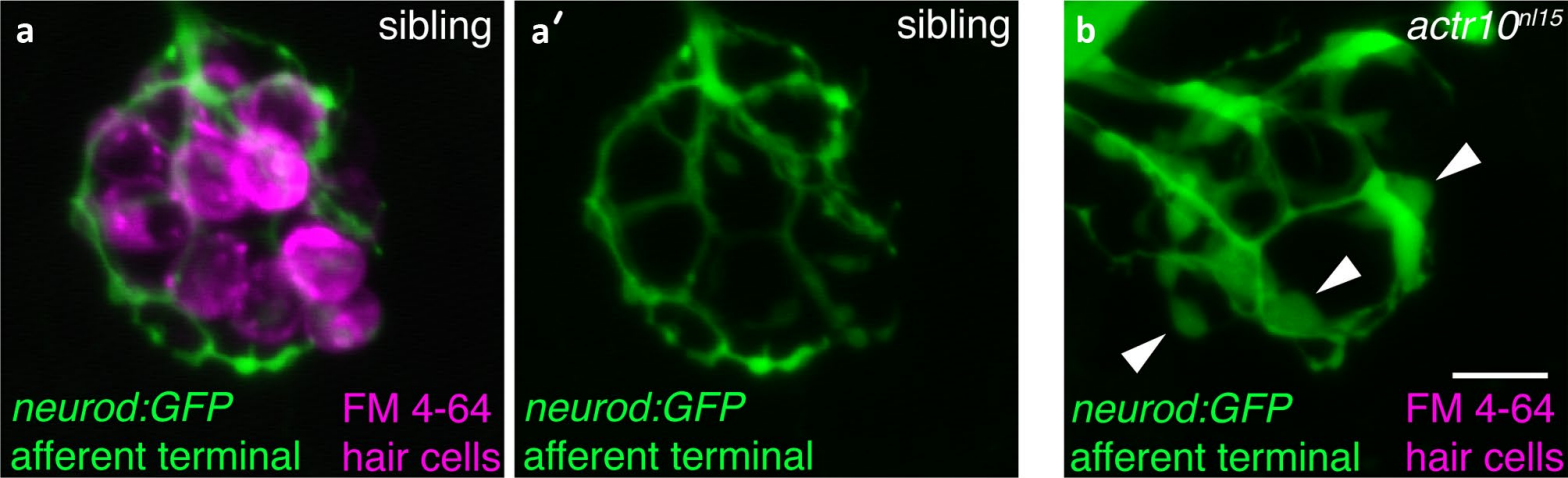
GFP-based forward genetic screen reveals mutants with swollen afferent terminals. (**a**–**b**) The *Tg(neurod1:EGFP)*^*nl1*^ transgenic line can be used to visualize the afferent terminals beneath hair cells in the lateral line (**a**–**b**, green), while the vital dye FM 4-64 can be used to visualize lateral line hair cells (**a**, **b**, magenta). *Actr10*^*nl15*^ mutants were isolated in a forward genetic screen using *Tg(neurod1:EGFP)*^*nl1*^ to identify mutants with swollen lateral line afferent terminals (**b**) (Drerup et al. 2017). Hair cells in these mutant do not label with FM 4-64. Arrowheads indicate swellings in **b**. Images were taken at 5 dpf at × 63 magnification. Scale bar in **b** = 5 µm

For example, a forward genetic screen was performed using the* neurod:GFP* transgenic line to phenotypically identify mediators of axonal transport in hair cell afferents (Fig. 1d′; Fig. 3a–b). The *neurod:GFP* transgenic line provides excellent in vivo labeling of the afferent cell bodies and the terminals that innervate hair cells in the lateral line and inner ear (Drerup and Nechiporuk 2013; Drerup et al. 2017; Obholzer et al. 2008). Mutants with defective axonal transport have swollen afferent terminals that can easily be visualized using the *neurod:GFP* transgenic line. Using this screening approach, several genes required for axonal transport have been identified (e.g., *actr10* (Fig. 3a–a′) and *jip3* (Drerup and Nechiporuk 2013; Drerup et al. 2017; Spinner et al. 2020)). One of the genes, *actr10*, is required for retrograde transport of mitochondria from the terminals innervating hair cells, back to the cell body (Fig. 3b). In *actr10* mutants, mitochondria accumulate in the afferent terminals, afferent terminals are swollen, and synaptic function is impaired (Mandal et al. 2021). Notably, the afferent terminal phenotypes and mitochondrial trafficking defects observed in *actr10* mutants may be related to the auditory neuropathy observed in Charcot–Marie–Tooth disease. This is a common hereditary neuropathy associated with impaired auditory processing. In this disease, mitochondria dysfunction has been implicated, but the precise mechanisms are not fully understood (Larrea et al. 2019; Rance et al. 2012)*.* Overall, this forward genetic screen for defects in axonal transport is notable because it was able to identify molecules necessary for the maintenance and function of hair cell afferents. In addition, this is an excellent example of a screen that is currently not possible mammals as sensory afferents cannot be visualized in toto.

Moving forward, there is an extraordinary potential to perform targeted forward genetic screens in live animals using transgenic zebrafish. These screens could identify molecules important for a wide variety of cellular functions relevant for hearing and balance. The *neurod:GFP* transgenic line could be used in conjunction with a transgenic line that labels hair cell presynapses (Fig. 1d, d′, *myo6b:ribeye b-mCherry* (Sheets et al. 2017)) to screen for molecules required for afferent neuron innervation and synapse formation. These same lines could also be used to identify molecules required to reform synapses after noise, or other excitotoxic agents that damage hair cell synapses or afferent neurons. There are numerous existing transgenic lines that could be used to study other aspects of anatomy required for hearing and balance. Similar screens could be performed to identify genes critical for the development of efferent neurons that innervate hair cells using transgenic lines that label the dopaminergic or cholinergic neurons, respectively (McCarroll and Nechiporuk 2013; Xi et al. 2011). Additionally, transgenic lines that label hair bundles could be used to reveal molecules required for hair bundle formation, morphology, or polarization (Fig. 1b–c′, *myo6b:actb1-GFP* (Kindt et al. 2012)). Overall, these types of morphology-based forward genetic screens using currently available transgenic lines could be used to identify molecular players important for a wide variety of cellular functions relevant for hearing and balance.

#### Behavior-Based Forward Genetic Screens to Identify Circler and Acoustic Startle-Deficient Mutants

Genes involved in hearing and balance have also been identified in forward genetic screens performed in larval zebrafish using assays based on behaviors dependent on hair cells systems. Zebrafish mutants with hearing and balance defects were initially identified as a distinct group of homozygous recessive motility mutants discovered in a large-scale forward genetic screen (Granato et al. 1996). The motility mutants in this distinct group were named “circler” mutants due to their balance-defective swimming behavior; they responded to tactile stimuli, but exhibited strong circling behavior when swimming, and failed to maintain an upright posture. The majority of the circler mutants also lacked an acoustic-vibrational startle reflex—a “fast start” escape response elicited by strong acoustic stimuli. In zebrafish, loss of this reflex can be roughly assessed by simply tapping on the petri dish in which the larvae are housed. This tap stimulus is thought to broadly stimulate the zebrafish auditory, vestibular, and lateral line systems, as well as somatosensory systems. Overall, this vibrational stimulus is a simple way to assay the function of hair cell systems in zebrafish (Nicolson et al. 1998). Later work using more specific auditory (pure tones generated by a vibration excitor (Einhorn et al. 2012; Erickson et al. 2017; Pacentine and Nicolson 2019; Smith et al. 2020)) and vestibular (vestibulo-ocular reflex (Mo et al. 2010)) assays confirmed that mutants initially identified using a tap stimulus had disrupted auditory and/or vestibular function. Importantly, despite these dramatic behavioral phenotypes, the majority of the circler mutants showed no major gross abnormalities with regard to inner-ear morphology (Granato et al. 1996; Nicolson et al. 1998).

Studies on mutant zebrafish with defects in hair cell systems (behavior and morphology) have largely been restricted to larval zebrafish. This is because mutants with strong circling behavior are not viable past 8 dpf (Nicolson et al. 1998). This inability to thrive has been attributed to a failure to inflate the swim bladder (an air-filled pocket that aids in buoyancy (Lindsey et al. 2010)) and circling behavior, which impairs motility and ultimately feeding in the 3-dimensional aquatic environment. Over the past 20 years, identification of the genetic lesions in zebrafish circler mutants has revealed many genes required for sensory-receptor function. For example, the first genetic lesion identified among the circler mutants was *myo7aa* (Ernest et al. 2000). *Myo7aa* zebrafish mutants have impaired sensory-receptor function; specifically, impaired hair cell mechanotransduction. Mutations in *myo7a* also result in hearing loss in humans (DFNB2 and DFNA11 Liu et al. 1997; Weil et al. 1995)) and mice (Gibson et al. 1995). This work and many subsequent studies in zebrafish, mice, and humans have shown that there is a remarkable conservation of genes required for sensory-receptor function in hearing and balance (e.g., *cdh23, pcdh15, myo6, myo7a, slc17a8, cacna1d, tmie, tomt, lhfpl5, dmxl2, get1* (Einhorn et al. 2012; Erickson et al. 2017; 2020; Ernest et al. 2000; Gleason et al. 2009; Lin et al. 2016; Obholzer et al. 2008; Seiler et al. 2004; 2005; Sidi et al. 2004; Söllner et al. 2004)).

#### Behavior-Based Forward Genetic Screens to Identify Genes Involved in Auditory Processing

Following these initial forward-genetic screens, additional behavioral screens in larvae were developed using robust and quantifiable properties of the acoustic startle response to identify mutants with deficits in auditory processing. These studies identified mutants that disrupt different aspects of the acoustic startle response including pre-pulse inhibition (PPI), contextual decision making, threshold sensitivity, and habituation (e.g., *cyfip2, casr*, *pappaa, pcxa,* and *ophelia* (uncloned) (Burgess and Granato 2007; Jain et al. 2011; 2018; Marsden et al. 2018; Wolman et al. 2015)). The first two genes identified using this quantitative approach were *pregnancy-associated plasma protein-aa* (*pappaa*), a metalloprotease involved in IGF receptor signaling, and *pyruvate carboxylase a* (*pcxa*), a rate limiting enzyme in glutamate production (Wolman et al. 2015). With regard to auditory processing, both *pcxa* and *pappaa* mutants have habituation defects. While wildtype zebrafish show reduced rates of startle or habituation after repeated acoustic stimuli (Wolman et al. 2011), extraordinarily, both *pcxa* and *pappaa* mutants fail to habituate to acoustic stimuli. Identifying and studying the function and cellular localization of these molecules required will be instrumental in understanding auditory processing and the underlying neural circuits.

Additional genetic approaches beyond classic forward genetic screens have helped cement zebrafish as a powerful system to study the circuitry underlying central auditory processing in zebrafish (Favre-Bulle et al. 2018; Marquart et al. 2019; Migault et al. 2018; Tabor et al. 2018; 2019; Vanwalleghem et al. 2017; 2020). For example, a novel circuit-breaking screen used chemogenetic ablation in larvae to “screen” for subsets of neurons important for modulation of the acoustic startle response. This type of circuit-breaking screen has identified specific populations of neurons that modulate acoustic startle onset (Tabor et al. 2018) and gating of PPI (Marquart et al. 2019). It is important to point out that analysis of mutants and neuronal subpopulations that impact central processing has benefited greatly by recent advances in whole brain imaging of activity and morphology. These imaging techniques have been used to map out brain regions that respond to distinct aspects of auditory, vestibular, and fluid flow stimuli in zebrafish (Favre-Bulle et al. 2018; Migault et al. 2018; Vanwalleghem et al. 2020). Together, robust behavior, defined neural circuits, and whole brain imaging now make zebrafish an impressive model system to dissect the genetic and neurological basis of hearing, balance and lateral line function.

#### Behavior-Based Forward Genetic Screens to Identify Genes Involved in Noise Exposure

In the future, behavior-based forward genetic screens could be performed in zebrafish to identify modulators that impact moderate and severe noise overexposure. Work in mice and zebrafish has shown that overexposure to noise can damage peripheral hair cells and innervating afferent neurons (Puel et al. 1998; Uribe et al. 2018; Holmgren et al. 2021). Additionally, moderately damaging noise exposures can also lead to perceptual abnormalities and change the behavioral response to sensory stimuli (Hickox and Liberman 2014; Šuta et al. 2015).

To date, several paradigms have been developed to expose larval zebrafish to damaging noise. In one study, to specifically target and damage the zebrafish auditory pathway, larvae were exposed to 18 h of moderately damaging, flat-spectrum noise at 20 dB (Bhandiwad et al. 2018). This study found that after this noise exposure paradigm, the threshold of acoustic startle responses was reduced by 10–15 dB. Additionally, noise-exposed larvae exhibited decreased habituation to startle-inducing stimuli, indicating a sensitized behavioral response. Auditory sensitivity, measured by PPI thresholds, did not change in noise-exposed larvae. Together, these results indicate after this damage paradigm, there is an increased gain in central neural excitability, rather than alterations or damage in the sensory organs of the ear. While mice have been used to screen for genes that modify noise-induced damage in the periphery (White et al. 2009), to our knowledge, there has not been a successful large-scale forward genetic screen for genes that modify central circuit function following noise damage. Therefore, the noise exposure paradigm developed in Bhandiwad et al. represents a valuable model to screen for genes that modify central circuit function.

Damage to the lateral line system in larval zebrafish has been delivered using two noise damage paradigms—one that induced mild to moderate damage specifically in the lateral line system, and a second that directed intense damage to both the inner ear and the lateral line system (Holmgren et al. 2021; Uribe et al. 2018). In the first paradigm, acute damage of the lateral line system was achieved using an electrodynamic shaker to deliver a 60 Hz, 41 m/s^2^ vibration stimulus for 2 h. This stimulus creates a strong water current that is sufficient to induce a fast escape response mediated by the lateral line system (Holmgren et al. 2021). Immediately following 2 h of overstimulation, lateral line hair cells showed reduced mechanotransduction and synapse loss. This damage began to recover within 2 h and was completely recovered within 48 h. In the second paradigm, more intense damage was achieved using 40-kHz ultrasonic transducers to generate small, localized shock waves to zebrafish larvae (Uribe et al. 2018). Exposure to this stimulus (165-dB, broadband noise) for 80 min induced delayed hair cell death that occurred 48–72 h post noise exposure. Hair cell death was observed in both the lateral line organs and in the utricular macula of the inner ear. Surviving hair cells in the lateral line showed signs of synapse loss but maintained normal mechanotransduction. Further, this study went on to demonstrate that hair cell death could be partially prevented by co-exposing larvae to antioxidants during noise exposure. Cumulatively, either of these two noise exposure paradigms could be used in a forward genetic screen to identify molecules involved in the effects of noise exposure in the periphery.

It is clear there is still a huge untapped potential for using forward genetic screens in zebrafish to explore the field of hearing and balance. Due to the time and scale involved, genetic screens in rodents are challenging because of the cost and the amount of breeding space required. In contrast, many zebrafish can be housed together inexpensively, making forward screens an attractive way to uncover novel molecules in an unbiased fashion. Comprehensively, future forward genetic screens could identify molecules that aid our understanding of inner ear function and development, hair cell regeneration and cell death, noise exposure, and ultimately auditory and vestibular behavior.

### Reverse Genetics to Study Hearing and Balance in Zebrafish

Sequencing of the mouse, human, and zebrafish genomes commenced in the 2000s, and in 2013, the first complete zebrafish genome sequence was published (Howe et al. 2013). Sequencing the zebrafish genome revealed an estimated 26,206 protein-coding genes, and a high degree of gene conservation between zebrafish and mammals. At the time of publication, the number of genes identified in the zebrafish genome was higher than any previously sequenced vertebrate genome. This increased gene number is largely due to a whole-genome duplication event in the ancestral past of the teleost lineage (Meyer and Schartl 1999). Duplicate genes in zebrafish have significant implications for forward genetic screens and investigating the role of molecules in hearing and balance. In some cases, gene duplicates evolved unique, non-overlapping functions. For example, this is true for *myo6* (DFNA22, DFNB37); *myo6b,* but not *myo6a*, is expressed specifically in zebrafish hair cells and is required for hearing and balance (Seiler et al. 2004). In other cases, gene duplicates have overlapping function and can act redundantly (Force et al. 1999; Kassahn et al. 2009). In cases when just a single zebrafish paralog is mutated, there may not be an obvious “screenable phenotype” (e.g., lack of an acoustic startle response or circling behavior) in a forward genetic screen (Amsterdam et al. 2011; Wu et al. 2018). Two examples of hearing and balance mutants that were not identified in zebrafish forward mutagenesis screens are *tmc1* (DFNB7/11) and *otof* (DFNB9). Recent work using targeted approaches to eliminate gene function revealed that two (*otofa*; *otofb*) or three (*tmc1*; *tmc2a*; *tmc2b*) paralogs respectively must be eliminated to abolish the acoustic startle response (Chatterjee et al. 2015; Chen et al. 2020; Smith et al. 2020).

To target and eliminate a known gene of interest, reverse genetic approaches have been developed and used in zebrafish. In contrast to classic forward genetics, where the genome is randomly mutagenized and phenotypes of interest are linked to novel genes, reverse genetic approaches disrupt the function of a known gene, then determine whether there are phenotypes associated with the disruption. Reverse genetic approaches can be used to transiently disrupt a specific gene product or mRNA. In larval zebrafish, this is most commonly done by using anti-sense morpholinos (Bill et al. 2009). Reverse genetics can also be employed at the genome level, through targeted lesions to a gene within the genome. To target the genome, mutagenic strategies (ENU and retroviral insertion) can be used followed by genome “screening” for lesions of interest (Amsterdam et al. 2011; Moens et al. 2008; Sood et al. 2006). More targeted approaches, using TALENS and CRISPR-Cas9, have dramatically enhanced the efficiency and ease of reverse genetics in zebrafish (Gaj et al. 2013; Hwang et al. 2013; Irion et al. 2014). Here we highlight several of these reverse genetic approaches including gene knockdown via morpholino, and knockout technologies (TILLING, CRISPR-Cas9) to target and assess the roles of genes in hearing and balance.

#### Using Morpholinos to Knockdown Gene Products Important for Hair Cell Systems

**Fig. 4:**
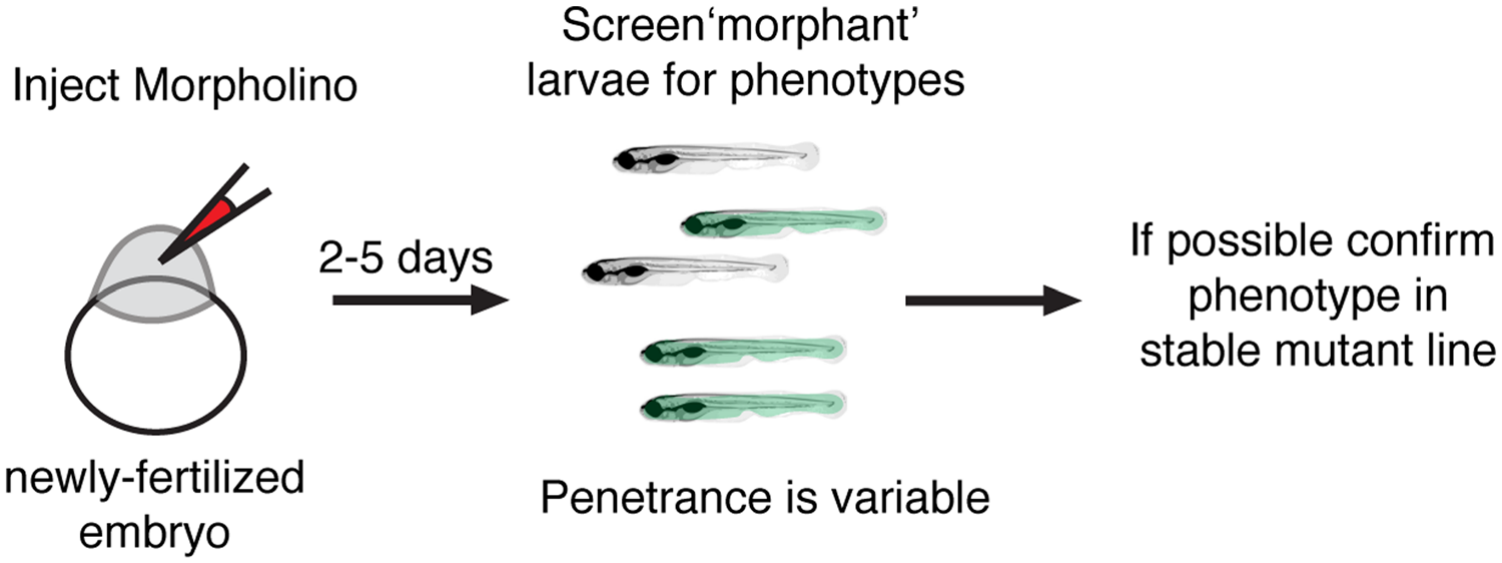
Outline of Morpholino timeline in zebrafish. Morpholinos (MOs) are used to block mRNA splicing or translation of a particular gene product. MOs are injected into newly fertilized zebrafish embryos. Two to 5 days after MO injection morphant larvae can be screened for phenotype. The penetrance of MO phenotypes is highly variable. It is recommended that MO phenotypes be confirmed using a germline mutant when possible (Stainier et al. 2017)

Concurrent with the onset of genome sequencing, methods to knockdown gene products were established (Fire et al. 1998; Weiss et al. 1999). Although not true genetic approaches, antisense approaches using RNA interference such as siRNAs (small interfering RNAs) became a powerful way to rapidly silence genes of interest (Nasevicius and Ekker 2000). Unfortunately, reliable siRNA-mediated gene knockdown techniques were never fully established in zebrafish; currently, there are only few conflicting reports that have demonstrated successful siRNA knockdown (Dodd et al. 2004; Kelly and Hurlstone 2011). Instead of siRNA, a related approach using antisense morpholino oligonucleotides (MOs) was developed in 2000 to knockdown gene products in developing zebrafish embryos (Fig. 4 (Nasevicius and Ekker 2000)). MOs are synthetic antisense oligos that function to either block mRNA translation by binding at the start site of mRNAs or by disrupting mRNA splicing at intron–exon junctions (Bill et al. 2009). MOs are fast—phenotypic screening can begin hours after they are injected into newly fertilized embryos. MOs are also resistant to degradation and persist in the developing larvae for several days. When used properly, MOs can be an effective way to knockdown a gene of interest and create “morphant” embryos and larvae for analyses of gene function.

Several studies have used MOs in zebrafish to successfully verify the identity of a candidate gene implicated in hair cell systems (Dutton et al. 2001a; Gleason et al. 2009; Lin et al. 2016; Obholzer et al. 2008; Söllner et al. 2004; Whitfield 2002). For example, MOs were used to help confirm that inner ear defects in zebrafish *colorless* mutants were due to a lesion in *sox10*, a gene associated with Waardenburg-Shah syndrome, an auditory-pigmentary disorder in humans (Bondurand et al. 2007; Dutton et al. [Bibr CR40][Bibr CR41]). Importantly, *sox10* morphants phenocopied *sox10* germline mutants—both had smaller otic vesicles and otoliths. In addition to these verification studies, MOs are often used in place of a germline mutant after the phenotype of the mutant has been determined (Blanco-Sánchez et al. 2014; Goodman and Zallocchi 2017; Trapani et al. 2009; Zhang et al. 2018). Using MOs can expedite experimental timelines. Instead of importing new mutant lines, MOs can simply be injected into embryos and used hours or days after injection. MOs have also been used extensively to probe the role of novel, previously uncharacterized genes that have been implicated in hearing and balance in humans (Azaiez et al. 2015; Delmaghani et al. 2016; Ebermann et al. 2010; Riazuddin et al. 2012; Yariz et al. 2012). For example, MOs were used in zebrafish to probe the role of TRRAP in hearing and balance (Xia et al. 2019). In this study, a novel, pathogenic variant in the *TRRAP* gene was identified within a human cohort associated with progressive hearing loss. In zebrafish *trrap* morphants there were fewer hair cells in each sensory epithelia and the acoustic startle responses were reduced. These phenotypes in zebrafish were also confirmed using a germline *trrap* zebrafish mutant.

Although effective at disrupting gene function, MOs present two main limitations to study some aspects of hearing and balance. The first issue is that MO concentration and overall effectiveness dilute with every cell division. MOs are injected at the 1 cell stage, into zygotes, and the subsequent phenotypes are assayed at a later stage of development. For early developmental phenotypes, loss of MO concentration through subsequent cell divisions is generally not a concern. MO knockdown has been extremely effective for molecules required early for placode formation, inner-ear morphogenesis, lateral line migration, and afferent neuron formation, all of which occur within the first 2 days of development (Andermann et al. 2002; David et al. 2002; Geng et al. 2013; Whitfield 2002). For example, similar to *neurog1a* germline mutants, *neurog1a* morphants fail to form hair cell afferent neurons (Andermann et al. 2002; López-Schier and Hudspeth 2005). This phenotype is permanent in morphants—after missing this developmental cell-fate milestone, afferents fail to form even when the MO is no longer present. However, some phenotypes, such as auditory and vestibular behaviors, are not fully established until 5 dpf (Bhandiwad et al. 2013; Zeddies and Fay 2005). At this stage, there is a higher probability that MOs have been diluted to the point that they no longer effectively disrupt mRNA translation, thereby leading to recovery of gene function (Bill et al. 2009; Timme-Laragy et al. 2012). Therefore, care needs to be taken to verify that MOs are still effective at later stages by using reagents to assay gene knockdown (via RT-PCR, qPCR or immunohistochemistry).

The second main issue that can arise with MOs use is the potential for off-target effects. Many discrepancies and concerns over specificity of MOs have been debated in recent years (El-Brolosy et al. 2019; Kok et al. 2015; Stainier et al. 2017). There are several reports indicating that in some instances, MOs can lead to the induction of p53-mediated cell death (Robu et al. 2007). This induction can lead to off target toxicity-related phenotypes that may not be related to knocking down of the actual protein of interest. MO-related toxicity has been shown in some instances to disrupt lateral line formation and gross development (Aman et al. 2011; Azuma et al. 2006; Ekker and Larson 2001). Therefore, the use of MOs requires strict guidelines to ensure that any observed phenotypes are due to loss of the targeted gene and not due to off-target or non-specific defects related to the reagent itself.

There are benefits to using MOs as well, particularly for early phenotypes. Some mRNA is deposited into the zygote by the female. In cases when an adult female heterozygous is used to generate mutant larvae, wildtype mRNA can be deposited and can persist through into larval stages in some instances. Early phenotypes in mutant animals will therefore be unobservable; however, MOs that target the start site of the gene can be used to disrupt the translation of these maternally deposited mRNAs. Additionally, there is concern for genetic compensation in CRISPR-induced mutant lines, which can lead to upregulation of gene homologs and mask phenotypes or at least phenotype severity (Buglo et al. 2020; El-Brolosy et al. 2019; Rossi et al. 2015). This genetic compensation, however, may be alleviated with MOs. Therefore, MOs, if used thoughtfully, can be a more effective tool for studying gene function in certain contexts.

Currently, the zebrafish community highly recommends that MO analyses be validated with an existing germline mutant obtained from ZIRC, or created using CRISPR-Cas9 (see below) (Stainier et al. 2017). If no stable mutant line can be obtained, there are several guidelines to follow: (1) validate phenotypes using multiple MOs, along with control MOs; (2) validate gene knockdown using RT-PCR, qPCR, or immunohistochemistry; (3) rescue the MO-associated phenotype with a mRNA not targeted by the MO and lastly; (4) be a robust experimentalist: perform a dose response curve, use a sufficient number of animals for statistical power, and employ blinding strategies (Stainier et al. 2017). Overall, despite the caveats that are associated with MO use, they remain a fast and powerful way to knockdown specific gene products in zebrafish.

#### Using TILLING and Retroviral Insertion Mutants to Study Hair Cell Systems

Until recently, two main approaches were used to identify germline zebrafish mutants in a gene of interest: retroviral (RV) insertion-based mutagenesis and Targeting Induced Local Lesions in Genomes (TILLING) (Kettleborough et al. 2013; Moens et al. 2008; Pan et al. 2015; Sood et al. 2006; Wienholds et al. 2003). RV-based mutagenesis leads to random insertion of viral sequences into the genome. TILLING utilizes DNA alkylating agents (e.g., ENU) to randomly induce single-nucleotide mutations in the genome. From these mutagenized animals (ENU or RV), a library of sperm is created. Sequencing of the sperm from this library yields a pool of potential mutants that can be recovered via in vitro fertilization. Both these approaches rely on random mutagenesis followed by sequencing to “screen” the genome of mutated fish for potentially pathogenic retroviral insertions or single nucleotide polymorphisms (SNPs) in a gene of interest (Fig. 2). Several groups, as well as the Zebrafish Mutation Project at Sanger, have used these approaches to create an immense collection of mutants for the community. Currently, there are over 37,000 TILLING alleles and 4000 RV insertion mutants (Varshney et al. 2013; the ZF mutation project). For example, the sole germline *neurog1a* mutant (*neurog1a*^*hi1059Tg*^, required for formation of hair cell afferent neurons, see above) contains a RV insertion (Golling et al. 2002). In addition, several mutant alleles of *get1*, a protein critical for zebrafish hearing and vision, were created by RV insertion and TILLING (Busch-Nentwich et al. 2013). The majority of these mutants are readily available from the Zebrafish International Resource Center (ZIRC), or the European Zebrafish Resource Center (EZRC). These collections of zebrafish mutants represent an invaluable asset for identifying and studying molecules required for hearing and balance. 

#### Targeted Genome Editing and Reverse Genetics Using CRISPR-Cas9

While the TILLING and RV insertion mutant collections are a valuable resource, they are not targeted. They rely on random chance and therefore do not cause mutations in all genes of interest or always result in detrimental genetic lesions. Current advances in reverse genetics using gene editing technologies, including Zinc-Finger Nucleases (ZFNs), Transcription Activator-like Effector Nucleases (TALEN), and Clustered Regularly Interspaced Short Palindromic Repeats (CRISPR)-Cas9, have dramatically changed the landscape of genetic research in the last decade (Gaj et al. 2013). CRISPR-Cas9 methods in particular have emerged as a favored approach to create targeted mutations in zebrafish and other model systems (Gasiunas et al. 2012; Hwang et al. 2013; Irion et al. 2014; Jinek et al. 2012; Mali et al. 2013).

**Fig. 5:**
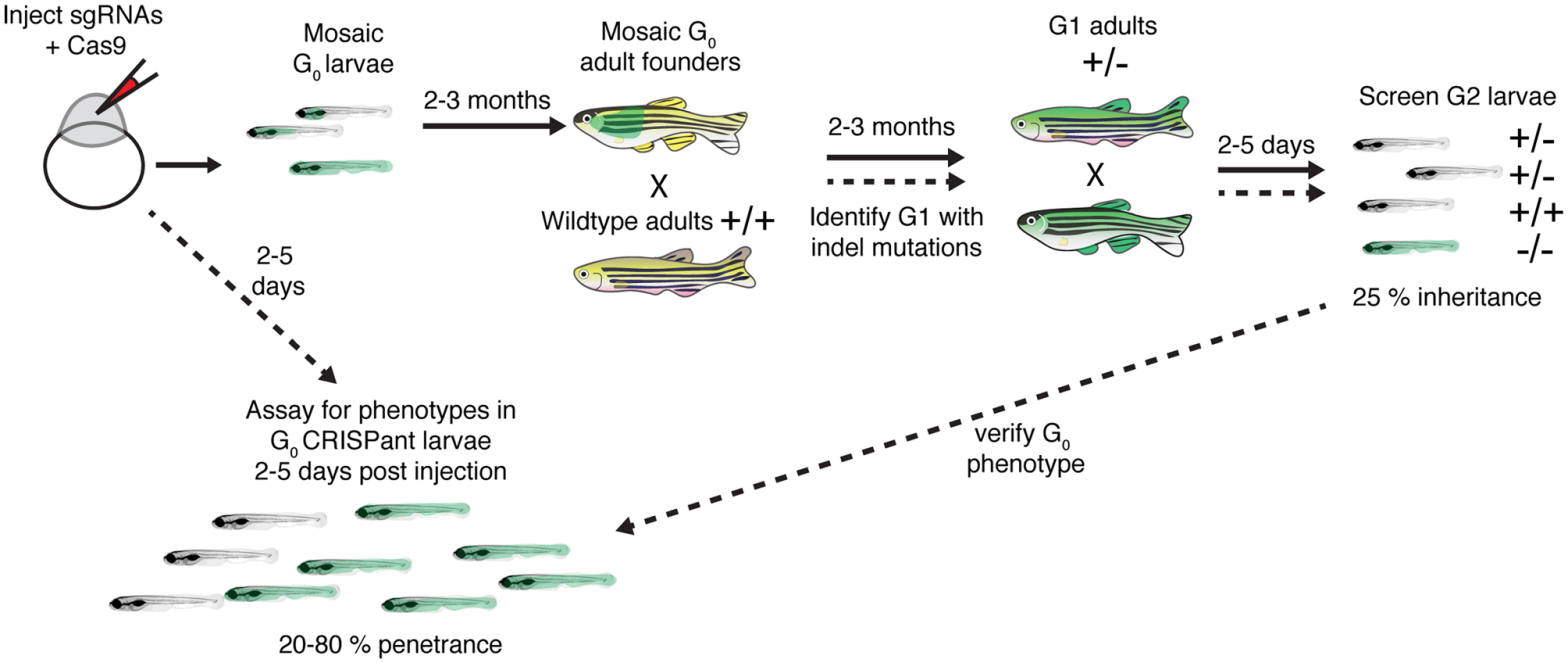
Outline of how to create germline zebrafish CRISPR-Cas9 mutants and CRISPants. To create a germline zebrafish CRISPR-Cas9 mutant (follow path of solid lines), guide RNAs (gRNAs) targeting a gene of interest along with Cas9 mRNA or protein are injected into newly fertilized zebrafish embryos. These G_0_ injected embryos are grown to adulthood (2–3 months). G_0_ adult founders are crossed to wildtype adults, and the G_1_ progeny screened for indels. G_1_ progeny with indels are grown to adulthood. G_1_ adults containing indels are then incrossed and screened for phenotypes. To perform analyses on injected G_0_ CRISPant larvae, optimized gRNAs are injected into newly fertilized zebrafish embryos. Injected CRISPant G_0_ larvae are then screened for phenotypes days after the injection. CRISPant phenotypes can be verified by generating a stable, germline mutant

In zebrafish, CRISPR-Cas9 methods are robust and relatively straightforward (Varshney et al. 2015); guide RNAs (gRNAs) along with either Cas9 mRNA or Cas9 protein are simply co-injected into newly fertilized embryos to target and induce a double stranded break at a specific genomic site (Fig. 5). Initial approaches to create loss of function CRISPR-Cas9 mutants in zebrafish have relied on errors in non-homologous repair at cut sites to create small insertions or deletions (INDELs) in a gene of interest. INDELs can shift the reading frame and lead to nonsense mediated decay or interfere with protein function (Irion et al. 2014). In addition to using CRISPR to target a single gene, it is also possible to target multiple genes or an entire gene family in a single set of zebrafish embryo injections (Jao et al. 2013; Kroll et al. 2021; Shah et al. 2015; Varshney et al. 2015). This “multi-plexing” makes it straightforward to target multiple zebrafish orthologs and paralogs at the same time. This high-throughput approach makes it easier to create double mutants and uncover new molecules that could not be identified phenotypically in forward genetic screens in cases where there is gene redundancy. Unfortunately, multi-plexing, although powerful, also has the potential for greater off-target effects (Dong et al. 2019; Wang et al. 2020). Therefore, is important to vigilantly confirm the relationship between the lesion and the phenotype.

In addition to creating new mutant models, CRISPR-Cas9 methods have been optimized to create biallelic mutations that enable detection of phenotypes in G_0_ CRISPR-Cas9 injected embryos or larvae (Burger et al. 2016; Hoshijima et al. 2019; Kroll et al. 2021; Swinburne et al. 2018). These CRISPR-Cas9 injected zebrafish are referred to as “CRISPants” or G_0_ knockouts. This technology is rapidly advancing—current studies have optimized this method of mutagenesis by ascertaining gRNAs effectiveness or using multiple gRNAs against a single gene target. These methods have been shown to efficiently produce a high percentage (20–90 %) of G_0_ embryos with mutations and associated phenotypes (Hoshijima et al. 2019; Kroll et al. 2021; Shah et al. 2015). For example, both CRISPant and germline mutants were used to study the role of *lmx1bb* in the zebrafish inner ear (Swinburne et al. 2018). This study found that the transcriptional factor *lmx1bb* is essential to form a pressure relief valve for fluid exchange in the endolymphatic sac, which is important for fluid homeostasis. Importantly, by utilizing CRISPants, this study was able to rapidly expedite several analyses.

While CRISPR-Cas9 has proved a straightforward way to disrupt gene function in zebrafish, this technology continues to evolve for use in more advanced genomic modifications such as knock-ins (Cornet et al. 2018; Liu et al. 2019; Prykhozhij and Berman 2018). The potential to facilitate additional, directed modifications to the genome using CRISPR-Cas9 holds real power for future genetic studies in zebrafish. To date, several approaches have used CRISPR-Cas9 knock-in approaches to engineer a specific mutation or insert DNA such as the coding sequence for GFP into enhancers or the open reading frame of genes. For these approaches, various repair templates are co-injected along with gRNAs and Cas9. For more precise genome editing (e.g., insert GFP in frame or create a specific SNP), the repair templates are flanked by homology arms to drive homology-directed repair (HDR) pathways for insertion (Bai et al. 2020; Zhang et al. 2016). Other, less precise approaches (e.g., insert GFP into an enhancer) rely on a more generic template and homology-independent repair for insertion (Auer et al. 2014; Kimura et al. 2014; Ota et al. 2016).

Evidence of the power of these knock-in approaches is already evident in studies of zebrafish hair cell systems. For example, one study used a knock-in approach to test the role of a specific variant (R180Q) in *slc9a3r,* a Na^+^/H^+^ exchange regulatory cofactor (Girotto et al. 2019). The R180Q variant was identified in a genome-wide association study (GWAS) in human patients affected by age-related hearing loss (ARHL) (Morgan et al. 2019). This study used a single-stranded oligonucleotide with two homologous arms along with HDR to engineer *slc9a3r1*^R180Q /R180Q^ zebrafish mutants. *Slc9a3r1* zebrafish mutants had smaller saccular otoliths (important for sound detection) indicating that the *SLC9A3R1* gene may be part of the pathology underling ARHL in human patients (Girotto et al. 2019). Another study used CRISPR-Cas9 knock-ins to insert either the photoconvertible fluorescent protein Eos or nitroreductase (NTR, for chemical ablation) into the enhancer region of genes expressed in distinct supporting cell populations in the lateral line system (Thomas and Raible 2019). These knock-ins enabled researchers to elucidate the identity and nature of progenitor populations during homeostasis and hair cell regeneration (Thomas and Raible 2019). More recently, another study used a similar approach (knock-in of GFP into an enhancer) to follow the lineage of Emx2, a transcription factor important for several cell-fate decisions in developing lateral line neuromasts (Ohta et al. 2020).

As CRISPR-Cas9 approaches become more efficient, they could be used in the future for more focused applications such as tissue specific knock-outs. Here, a knock-in approach could be used to flank a deafness-associated gene with loxP sites. These knock-ins could be used along with transgenic lines that express Cre recombinase in specific cell types (e.g., transgenic lines that express Cre in hair cells or primary afferents) (Almeida et al. 2021; Burg et al. 2018; Li et al. 2019). In addition, CRISPR-Cas9 knock-ins could be applied to fluorescently tag molecules at their endogenous locus (DiNapoli et al. 2020), in order to observe protein function, localization, and dynamics in vivo. Overall, the ability to create zebrafish knock-ins will greatly enhance the ability to define molecular function of critical deafness genes.

### Using Zebrafish High-throughput Reverse Genetics to “Screen” Genomic and Transcriptomic Data

Next-generation sequencing continues to generate a fast-moving front of data at the genomic and the transcriptomic level. At the genome level, both whole-genome and exome sequencing continue to increase the identification of genes associated with human hearing loss (Chen et al. 2015; Erickson et al. 2017; Gao and Dai 2014; Girotto et al. 2019; Lin et al. 2012; Morgan et al. 2019; Ryu et al. 2017; Vona et al. 2014; Wells et al. 2019; Yan et al. 2013). Concurrently, at the transcriptome level, RNA-sequencing (RNAseq) and single-cell RNA-sequencing (scRNA-seq) continue to identify new genes expressed during development, regeneration or pathology (Barta et al. 2018; Ealy et al. 2016; Kolla et al. 2020; Liu et al. 2020; Scheffer et al. 2015) and to identify new genes linked with individual cell types within hair cell sensory systems (Barta et al. 2018; Burns et al. 2015; Cheng et al. 2017; Liu et al. 2014; Lush et al. 2019; Petitpré et al. 2018; Yizhar-Barnea and Avraham 2017). In zebrafish specifically, there are several transcriptomic datasets related to hair cell systems (Erickson and Nicolson 2015; Matern et al. 2018; Steiner et al. 2014). Several of these datasets are hosted on the gEAR database (https://umgear.org) or hosted on alternatives sites ((Lush et al. 2019) https://piotrowskilab.shinyapps.io/neuromast_homeostasis_scrnaseq_2018).

With these advances and this wealth of information comes the need for robust and high-throughput methods to characterize this new genetic data. Unfortunately, without a viable way to verify or evaluate the role of these gene mutations, genetic variants, or gene expression profiles, this wealth of information quickly becomes noise. Currently, the information generated in these studies has been verified using in situ hybridization or immunohistochemistry approaches. But the most compelling way to verify this databank of genetic information is to examine morphological and functional changes in mutants with a lesion in a gene of interest.

#### Applying Reverse Genetics to Study “Omics” Datasets Derived from Hair Cell Systems

In an ideal scenario, a genomic or transcriptomic dataset will yield an obvious gene target for follow-up analyses. This was the case for a transcriptomic study that examined the mRNA transcripts present in the adult zebrafish ear after regeneration following acoustic trauma (100 Hz tone at 179 dB re 1 μPa for 36 h) (Schuck et al. 2011). This transcriptomic dataset, generated using a microarray analysis, found that growth hormone (*gh1*) was the most upregulated gene (64.4 fold) during regeneration following acoustic trauma. The researchers in this study also used qRT-PCR to validate that *gh1* and other mRNAs were up- and down-regulated in their microarray study. Although this validation was an important control, using reverse genetics to create mutants, it is now possible to examine the role of molecules identified in this and other related studies on hair cell regeneration. Using reverse genetics to validate hair cell regeneration datasets has the potential to shed light on the differences between mammals and zebrafish in their regenerative abilities.

A recent study showed the feasibility of using zebrafish mutants to screen a transcriptomic dataset with multiple gene targets, in a relatively high-throughput manner. This work started with a dataset that isolated mRNA transcripts from the adult zebrafish inner ear during regeneration following acoustic trauma (100–10,000 Hz, 150–170 dB re 1 μPa for 48 h) (Liang et al. 2012). These mRNA transcripts were used in a later study to guide a reverse genetic screen. For this work, existing zebrafish mutants (RV-insertion mutants, see above section) were combined with germline CRISPR-Cas9 mutants that were created de novo. The screening platform assessed whether mutants had a hair cell regeneration phenotype (Pei et al. 2018). Overall, a total 254 germline zebrafish mutants were tested and 7 were confirmed to impact hair cell regeneration. This study demonstrates that the zebrafish model is a powerful high-throughput screening tool for defining the functional roles of genes from transcriptomic datasets relevant to hearing and balance.

#### High Throughput G_0_ CRISPant Screening for Hearing and Balance: a Test

The work by Pei et al. (2018) highlighted that using the zebrafish model can be an effective tool to evaluate gene function in a semi-high throughput way. Compared to the expense of MOS ($400 per MO), CRISPR-Cas9 approaches in zebrafish using G_0_ CRISPant screens can be used to quickly and inexpensively phenotypically test the role of candidate genes. Moreover, by simply growing injected larvae, germline zebrafish mutants can be used to confirm relevant phenotypes identified in pilot screens.

**Fig. 6:**
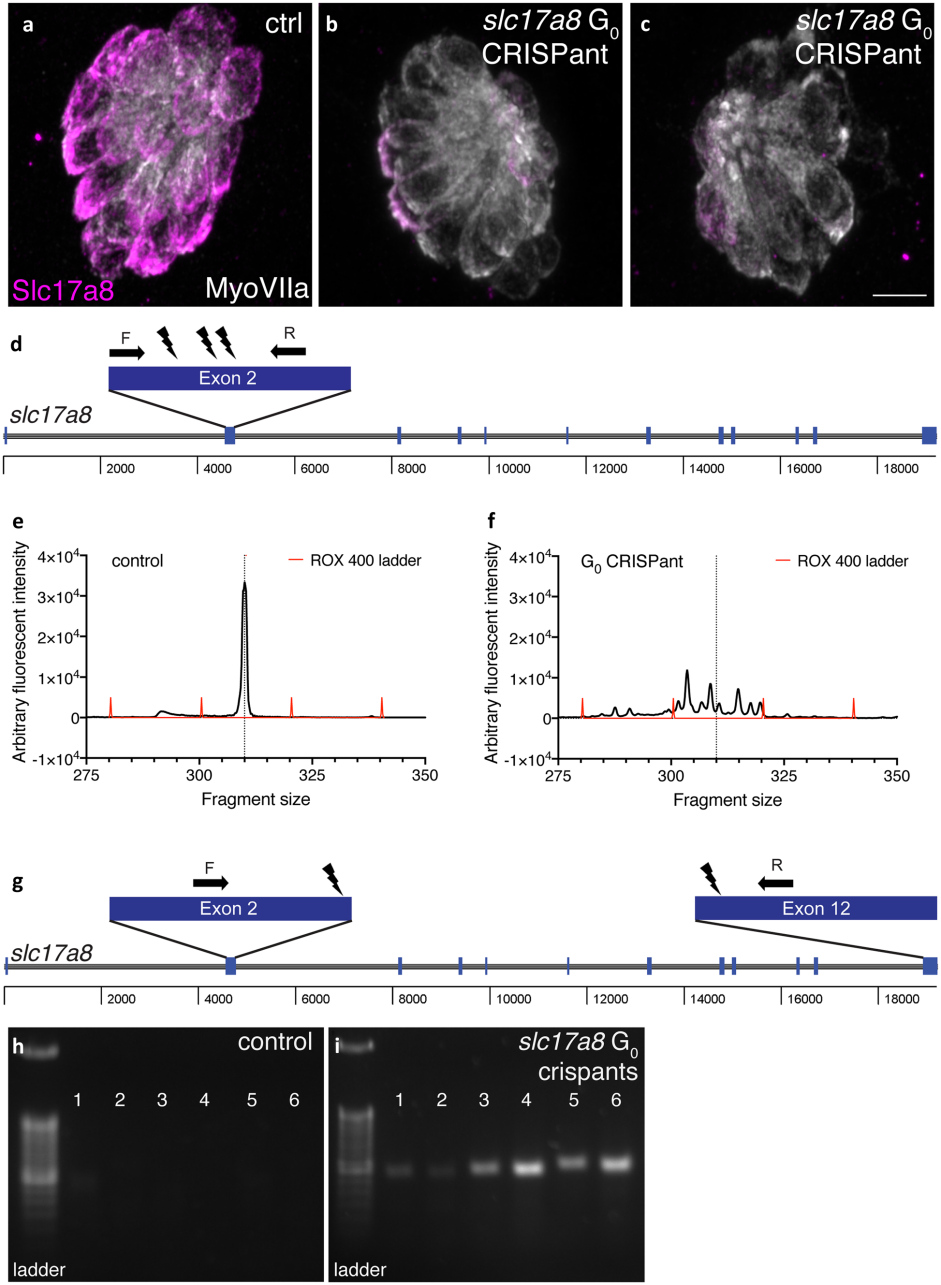
Two examples G_0_ *slc17a8* CRISPant analysis and genotyping. Neuromasts from uninjected **a** and CRISPants embryos injected with the following gRNAs directed against the following sites in exon 2 of *slc17a8* (5′-3′): GACAGAAGATGGTCGGCCGG (TGG), GGTGCTTTGGCCTTCCCAAA (CGG), and GCCCACCCCTATTGGACTGT (GGG) along with Cas9 protein **b**–**c.** Staining with anti-Slc17a8 (Obholzer et al. 2008) and anti-MyosinVIIA (Developmental Studies Hybridoma Bank, #138-1) to label lateral line hair cells reveal that Slc17a8 staining is absent in G_0_ CRISPants that lack an acoustic startle response. Schematic of PCR analysis of *slc17a8 ***d** used to detect INDELs. The CRISPR-STAT assay, relying on fluorescent fragment analysis can be used to genotype individual CRISPants larvae and test gRNA efficiency. In these examples, there is a single peak in control larvae at 310 bp **e**. By comparison, in G_0_ *slc17a8* CRISPants the peak at 310 bp is degraded, and numerous fragments (indicative of the many INDELs present in this mosaic founder) surrounding this peak are present **f**. Schematic of PCR analysis of *slc17a8* **g** used to detect a large deletion. This PCR analysis was conducted on genomic DNA from uninjected control and CRISPant larvae lacking a startle response. Primers flank the sites targeted by the guides targeting exon 2 ((5′-3′)CACAGTCTACATCAACGGGA(CGG)) and exon 12 (TCCAGTGTAATGCACCATGG(AGG)) and were used to amplify the region between exon 2 and exon 12. Deletion of a 14.2-kb region in CRISPants yielded an ~ 400-bp PR product (lanes 1–6, **i**) that was absent in uninjected controls (lanes 1–6, **h**). Images in **a**–**c** were taken at × 63 magnification on a Zeiss LSM 780 confocal microscope. Scale bar in **c** = 5 µm

As proof of principle, our two labs assessed the potential power of the G_0_ CRISPant approach for studies of hearing and balance by independently designing and injecting gRNAs against *slc17a8* (Fig. 6). Slc17a8 is critical for hair cell neurotransmission and hearing and balance in zebrafish (Obholzer et al. 2008). In one lab, 3 gRNAs targeting exon 2 were injected into embryos to generate INDELs in exon 2 (Fig. 6d). In the other lab, two gRNAs, one in exon 2 and one in exon 12, were used to generate a large deletion (approach described in Hoshijima et al. 2019; Fig. 6g). Using both targeting approaches, we found that just 5 days after injection ~ 80 % of G_0_ CRISPant larvae lacked an acoustic startle response. In addition, in *slc17a8* CRISPants lacking an acoustic startle response, we observed a dramatic reduction of Slc17a8 immunolabel in hair cells compared to controls (Fig. 6a–c). Based on the type of genetic lesions produced in the two approaches, we also used two different methods to verify mutagenesis in individual *slc17a8* CRISPant larvae.

To detect CRISPants with INDELs in exon 2, we used a fluorescent-polymerase chain reaction (f-PCR)-based method called CRISPR Somatic Tissue Activity Test (CRISPR-STAT) (Carrington et al. 2015). For CRISPR-STAT, a capillary sequencer is used to detect the size of the f-PCR products; the products are read out as peaks in a fragment analysis. In control individuals, the f-PCR amplification of the mutagenized region is visualized as a single peak in fragment analysis (Fig. 6e). In contrast, after effective mutagenesis in a G_0_ CRISPant larvae, multiple smaller peaks or fragments (representing various INDELs) are detected (Fig. 6f). To detect G_0_ CRISPants with a large deletion created by with sgRNAs targeting exon 2 and 12, we used PCR that revealed a gel band if both gRNAs successfully cut their genomic targets (Fig. 6i vs. Fig. 6h). Both of these approaches allowed us to verify effective mutagenesis in individual *slc17a8* CRISPant larvae. Overall, these examples independently demonstrate that CRISPR-Cas9 mutagenesis can be used to rapidly and effectively to assess gene function in G_0_ larvae.

## CONCLUSIONS AND OUTLOOK (FIG. 7)

**Fig. 7:**
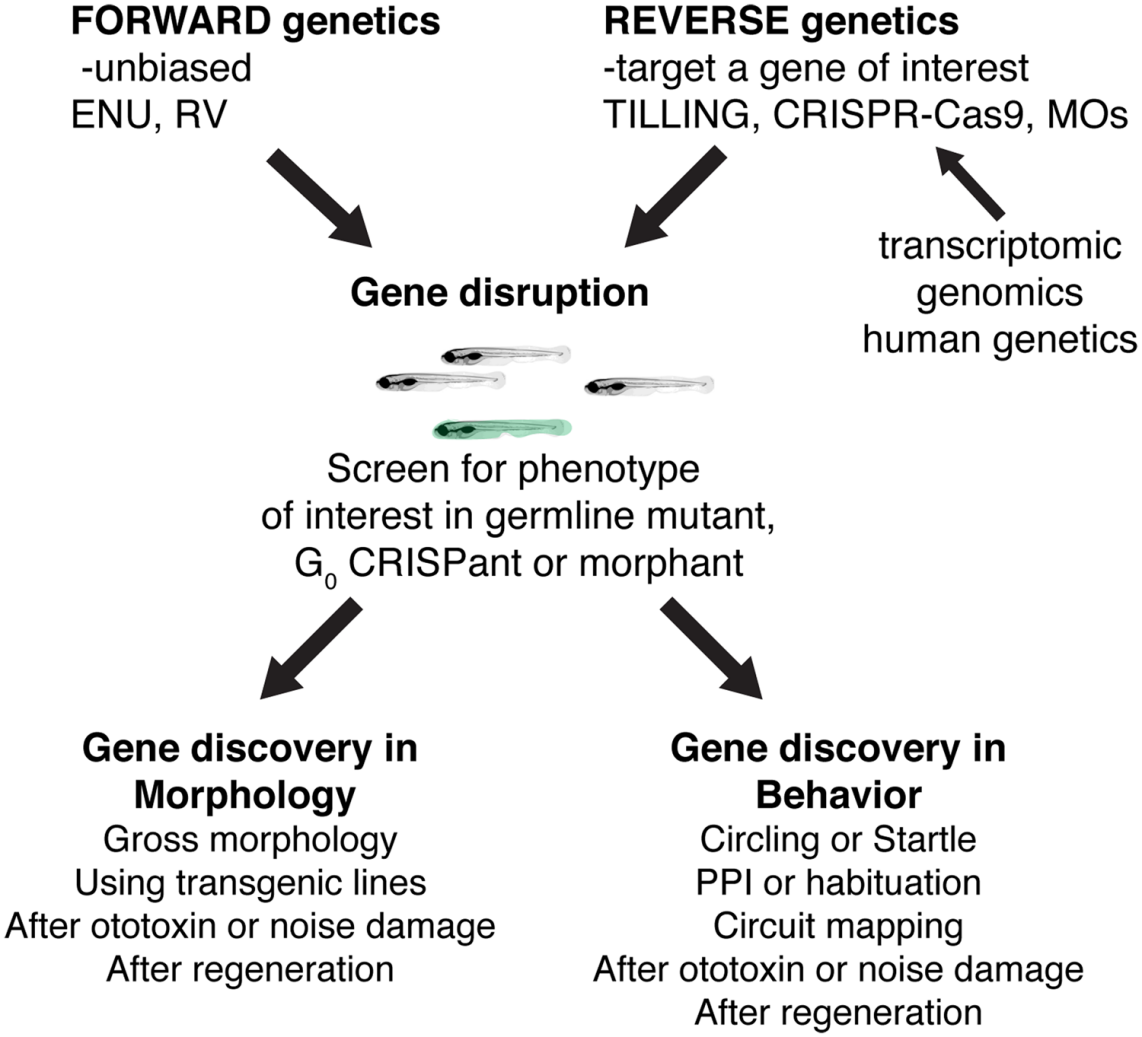
Past, present, and future ways to use zebrafish genetics to study hearing and balance. Both forward and reverse genetic approaches in zebrafish have had an immense impact on gene discovery in hearing and balance. In the future, novel forward genetic screens using transgenic lines or novel damage paradigms have the potential to continue this path to gene discovery. In addition, advances in reverse genetics will continue to provide a valuable way to screen genes implicated in humans hearing loss. Reverse genetic screening many also prove a valuable, high-throughput pipeline to validate transcriptomics or genomics datasets

The identification of mutants in zebrafish forward genetic screens uncovered highly conserved genes essential for hair cell sensory system development and function. These screens thereby established zebrafish as a valuable vertebrate model system to study hearing and balance. Over the past 20 years the advantages of the zebrafish model—fast and observable development, optical and pharmacological accessibility, and the ability to regenerate complex tissues—have been enhanced by the numerous stable transgenic and mutant lines available to researchers. In the future, genetic screens using zebrafish have the potential to identify not only additional genes important for hearing and balance but also define the molecular pathways contributing to damage and repair following toxic stimuli. Furthermore, using reverse genetics, it is now possible to engineer specific mutant alleles in zebrafish to mimic genetic lesions identified in humans. These lesions can then be characterized in the zebrafish model, providing in-depth information on protein function at the cellular level. Finally, genomic and transcriptomic studies continue to provide a wealth of genetic information. The zebrafish model provides a valuable tool to validate “omics” studies using CRISPR-Cas9 in G_0_ larvae to test hypotheses on gene function.

## Data Availability

All data is available on request.

## Code Availability

No coding data is associated with this work.
